# Dysregulation and imbalance of innate and adaptive immunity are involved in the cardiomyopathy progression

**DOI:** 10.3389/fcvm.2022.973279

**Published:** 2022-09-06

**Authors:** Bin He, Li-Ping Quan, Chun-Yu Cai, Dian-You Yu, Wei Yan, Qin-Jiang Wei, Zhen Zhang, Xian-Nan Huang, Li Liu

**Affiliations:** ^1^Graduate School of Youjiang Medical University for Nationalities, Baise, China; ^2^Department of Cardiology, Affiliated Hospital of Youjiang Medical University for Nationalities, Baise, China; ^3^Department of Cardiology, Affiliated Hospital of Youjiang Medical University for Nationalities, College of Clinical Medicine, Youjiang Medical University for Nationalitie, Baise, China

**Keywords:** cardiomyopathy, weight gene co-expression network analysis (WGCNA), biomarkers, immune cell infiltration, LASSO regression

## Abstract

**Background:**

Cardiomyopathy is known to be a heterogeneous disease with numerous etiologies. They all have varying degrees and types of myocardial pathological changes, resulting in impaired contractility, ventricle relaxation, and heart failure. The purpose of this study was to determine the pathogenesis, immune-related pathways and important biomarkers engaged in the progression of cardiomyopathy from various etiologies.

**Methods:**

We downloaded the gene microarray data from the Gene Expression Omnibus (GEO). The hub genes between cardiomyopathy and non-cardiomyopathy control groups were identified using differential expression analysis, least absolute shrinkage and selection operator (LASSO) regression and weighted gene co-expression network analysis (WGCNA). To assess the diagnostic precision of hub genes, receiver-operating characteristic (ROC) curves as well as the area under the ROC curve (AUC) were utilized. Then, Kyoto Encyclopedia of Genes and Genomes (KEGG) enrichment pathway analysis and Gene Ontology (GO) analysis were conducted on the obtained differential genes. Finally, single-sample GSEA (ssGSEA) and Gene Set Enrichment Analysis (GSEA) were utilized to analyze the infiltration level of 28 immune cells and their relationship with hub genes based on gene expression profile data and all differential gene files.

**Results:**

A total of 82 differentially expressed genes (DEGs) were screened after the training datasets were merged and intersected. The WGCNA analysis clustered the expression profile data into four co-expression modules, The turquoise module exhibited the strongest relationship with clinical traits, and nine candidate key genes were obtained from the module. Then we intersected DEGs with nine candidate genes. LASSO regression analysis identified the last three hub genes as promising biomarkers to distinguish the cardiomyopathy group from the non-cardiomyopathy control group. ROC curve analysis in the validation dataset revealed the sensitivity and accuracy of three hub genes as marker genes. The majority of the functional enrichment analysis results were concentrated on immunological and inflammatory pathways. Immune infiltration analysis revealed a significant correlation between regulatory T cells, type I helper T cells, macrophages, myeloid-derived suppressor cells, natural killer cells, activated dendritic cells and the abundance of immune infiltration in hub genes.

**Conclusion:**

The hub genes (CD14, CCL2, and SERPINA3) can be used as markers to distinguish cardiomyopathy from non-cardiomyopathy individuals. Among them, SERPINA3 has the best diagnostic performance. T cell immunity (adaptive immune response) is closely linked to cardiomyopathy progression. Hub genes may protect the myocardium from injury through myeloid-derived suppressor cells, regulatory T cells, helper T cells, monocytes/macrophages, natural killer cells and activated dendritic cells. The innate immune response is crucial to this process. Dysregulation and imbalance of innate immune cells or activation of adaptive immune responses are involved in cardiomyopathy disease progression in patients.

## Introduction

Cardiomyopathies are a diverse set of cardiac muscle illnesses characterized by electrical or mechanical abnormalities, typically exhibiting abnormal ventricular dilation or hypertrophy, thus contributing to the decline in systolic and diastolic function in heart failure (1). The etiology of cardiomyopathy is diverse, and its classification varies by country. For example, cardiomyopathy is classified according to etiology in the American Heart Association classification, while the European Society of Cardiology classification is based on a combination of morphology and hemodynamics. Currently, cardiomyopathy is mainly divided into primary and secondary cardiomyopathies. Primary cardiomyopathy can be classified as hereditary, such as arrhythmogenic right ventricular cardiomyopathy (ARVC), hypertrophic cardiomyopathy (HCM), acquired, such as inflammatory cardiomyopathy (ICM), and mixed, such as hereditary and acquired dilated cardiomyopathy (DCM). Secondary cardiomyopathy mainly includes alcoholic, ischemic, and perinatal cardiomyopathy (2, 3). Currently, it is not clear whether cardiomyopathy of different etiologies involves common mechanisms in molecular genetics changes or pathogenesis. Though, understanding these mechanisms is critical for managing and treating cardiomyopathy. However, to the best of our knowledge, we are innovatively combining cardiomyopathy with various etiologies to explore the key genetic changes or pathogenesis of cardiomyopathy progression compared to non-cardiomyopathy individuals by bioinformatics method.

The development of various phenotypes in cardiomyopathy depends on the complex interactions between individual genetic genotypes, multiple cellular signaling pathways, and environmental stressors. Although the pathogenesis of cardiomyopathy varies by etiology, inflammation and the immune system both play important roles in mediating irreversible damage to the myocardium (4, 5). When the heart is damaged or stressed, innate immune cells, such as neutrophils and monocytes, will migrate to the damage site and release mediators such as reactive oxygen species (ROS) and proteases to remove the factors that cause heart damage. However, after injury, Cardiomyocytes will further secrete pro-inflammatory cytokines that can trigger adaptive immunity and aggravate the inflammatory response (6). Current studies have shown that myocardial inflammation involves multiple inflammatory pathways, such as the TNF/NF-κβ pathway associated with cardiac infection and injury, pattern recognition receptors expressed by macrophages such as Toll-like receptors (TLRs), and oxidative and stress-activated caspase-1 inflammasome pathway and so on (7). In conclusion, inflammation plays a vital function in cardiomyopathy progression and pathogenesis. Therefore, the regulation of inflammation remains a promising target for treating cardiomyopathy of different etiologies. To find out the key immune-related pathway involved in them, our study further elucidates the inflammatory infiltration mechanism of cardiomyopathy.

In this study, we merged and intersected the data from cardiomyopathy groups with various etiologies so that the differentially expressed genes (DEGs) obtained by screening were more representative. Next, we intersected DEGs with the candidate key genes determined using weighted gene co-expression network analysis (WGCNA). Following this, the hub genes that distinguish the cardiomyopathy group from the non-cardiomyopathy control group were screened out using the least absolute shrinkage and selection operator (LASSO) regression on the basis of the intersection genes. Combining these methods increases the accuracy of the targeted signature genes for screening. Most importantly, we validated the screened hub genes expression level and diagnostic ability in the cardiomyopathy and non-cardiomyopathy control group in a large independent sample dataset (validation group). In addition, we conducted KEGG pathway enrichment analysis, Gene Ontology (GO) on DEGs, and Gene Set Enrichment Analysis (GSEA) on all differential gene files to identify their inflammatory and immune-related signaling pathways. Finally, using single-sample GSEA (ssGSEA), we investigated the infiltration of 28 immune cells based on expression profile data and their connection with hub genes. The current study would help us understand the cardiomyopathy pathogenesis and identify novel predictive and treatment targets for cardiomyopathy.

## Materials and methods

### GEO data download

We downloaded the Microarray expression data from the Gene Expression Omnibus (GEO) (http://www.ncbi.nlm.nih.gov/geo/) (8). The dataset for this analysis is divided into validation and training datasets. The training dataset included the following: GSE42955 (9) (12 cases of DCM, 12 cases of ICM, and five cases of controls), where GPL6244 platform of Affymetrix Human Gene 1.0 ST Array served as the foundation, in addition to GSE29819 (10) (12 cases of ARVC, 12 cases of DCM, and 12 cases of controls), where GPL570 platform of Affymetrix Human Genome U133 Plus 2.0 Array served as the foundation. The 17 controls of the training dataset (GSE42955 and GSE29819) were derived from non-diseased donor hearts, which could not be transplanted for technical reasons. The large sample dataset was used as the validation dataset: GSE5406 (11) (86 DCM, 108 ICM, and 16 controls), where GPL96 platform of Affymetrix Human Genome U133A Array served as the foundation. The 16 controls of the validation dataset (GSE5406) were from non-diseased normal hearts that had normal left ventricular function. The training and validation datasets can be subdivided into cardiomyopathy (Treat) and non-cardiomyopathy control (Con) groups.

### Data merging, intersecting, and screening of DEGs

The data from GSE42955 and GSE29819 datasets were merged and intersected using “sva” and “limma” R software packages (version 4.2.0), and the data were probe-annotated, batch-corrected, and normalized. Probe annotation files provided by researchers were employed to translate probes in each dataset into gene symbols. We determined the amount of gene expression in a given tissue by using the average numbers of probes that correspond to the same gene symbol. A systematic evaluation of ComBat's performance demonstrates that it outperforms other tools. Therefore, we used ComBat to eliminate batch effects between the two datasets (12). Both “pheatmap” package and “ggplot2” package were deployed to create DEGs heatmaps and volcano plots, respectively (13). The screening criteria of DEGs were customized to log fold change (FC) > 1 and *P* < 0.05 (14, 15).

### Building gene co-expression networks and finding the most relevant modules for clinical traits

From the expression profiling data of the merged datasets, a weighted gene co-expression network was created using the WGCNA package of the R program (16). We used the “goodSampleGenes” function to check the data's integrity and the “pickSoftThreshold” function to verify the ideal soft threshold (β) to correlate to a scale-free network more closely. After obtaining the matrix data, we converted it into a topological overlap matrix, and then gene clustering was performed, and the clustering results were identified by dynamic shearing module. Next, a hierarchical clustering dendrogram was built after calculating the module eigengenes (MEs) and combining related modules in the clustering tree based on the MEs. The modules were subsequently merged with phenotypic data to generate heatmaps. Subsequently, the correlations between genes and modules (MM) and the importance of genes (GS) were calculated. Finally, correlation histograms and scatter plots were drawn according to the MM and GS values.

### Hub gene screening, expression level verification, and diagnostic ability of the hub gene

According to the screening criteria (absolute value of MM > 0.80, absolute value of GS > 0.50), candidate key genes were picked from the modules with the greatest connectivity. Using R's “venn” package, we intersected the candidate key genes with the DEGs. Lastly, the final hub gene was screened by LASSO regression (17). The expression levels of the obtained hub gene in the training datasets were compared between the control group and the cardiomyopathy group using a boxplot, which was validated on an independent, large-sample dataset (GSE5406). The accuracy of the hub gene as a marker gene for cardiomyopathy and control groups was also assessed using the Receiver-operating characteristic (ROC) curve, and the diagnostic ability was further validated in the validation dataset.

### GO, KEGG, and GSEA functional enrichment analysis

KEGG enrichment analysis and GO were conducted on DEGs using the “clusterProfiler” and “enrichplot” packages of the R software (18). For all differential gene files, we used the immune-related gene sets that we obtained from the Molecular Signature Database (MsigDB) for GSEA enrichment (19). The top five significantly enriched immune gene sets were displayed. Considered statistically significant P values were adjusted at <0.05 (q <0.05).

### ssGSEA enrichment analysis to assess immune cell infiltration on profile data expression and its association with hub genes

With the ssGSEA algorithm, we assessed the correlation of gene expression profiles with the 28 immune cells (20). The differential expression levels of 28 immune infiltrating cells in the cardiomyopathy and non-cardiomyopathy control groups were visualized using Violin plots and heatmaps. The degree of association between the 28 immune cells and hub gene was evaluated by Spearman correlation and visualized using R software's “ggplot2” package.

## Results

### WGCNA analysis for the construction of a co-expression network for the identification of important modules and genes

**Figure 1:**
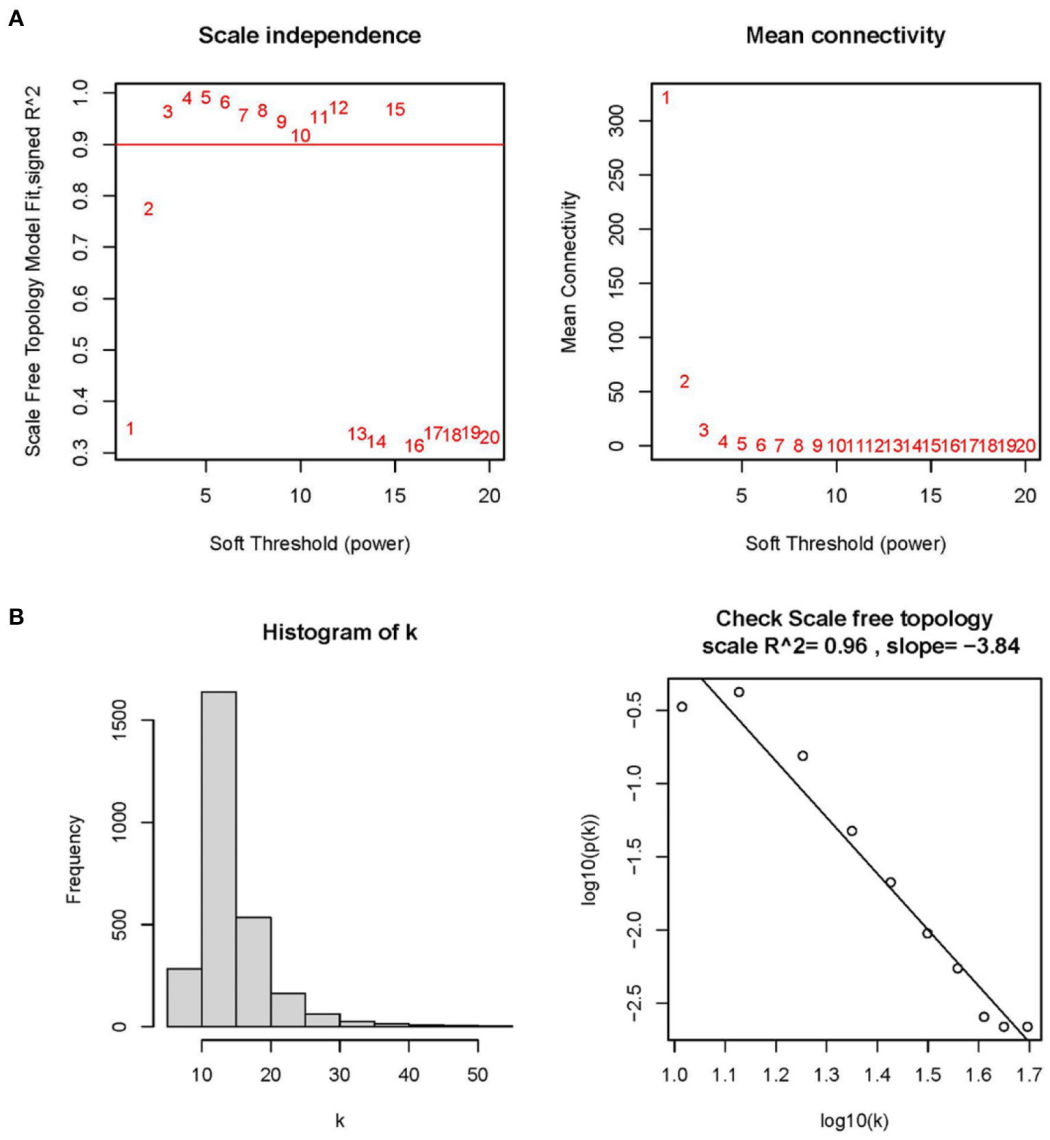
Determining optimal soft thresholds (β) in WGCNA. **(A)** Examination of the average connectivity under various β and scale-free fitting index. The red line implies that the corresponding soft threshold is 10 when the correlation coefficient is 0.9. **(B)** Connectivity distribution histogram and a scale-free network correlation coefficient of 0.96 checked at β = 3.

**Figure 2:**
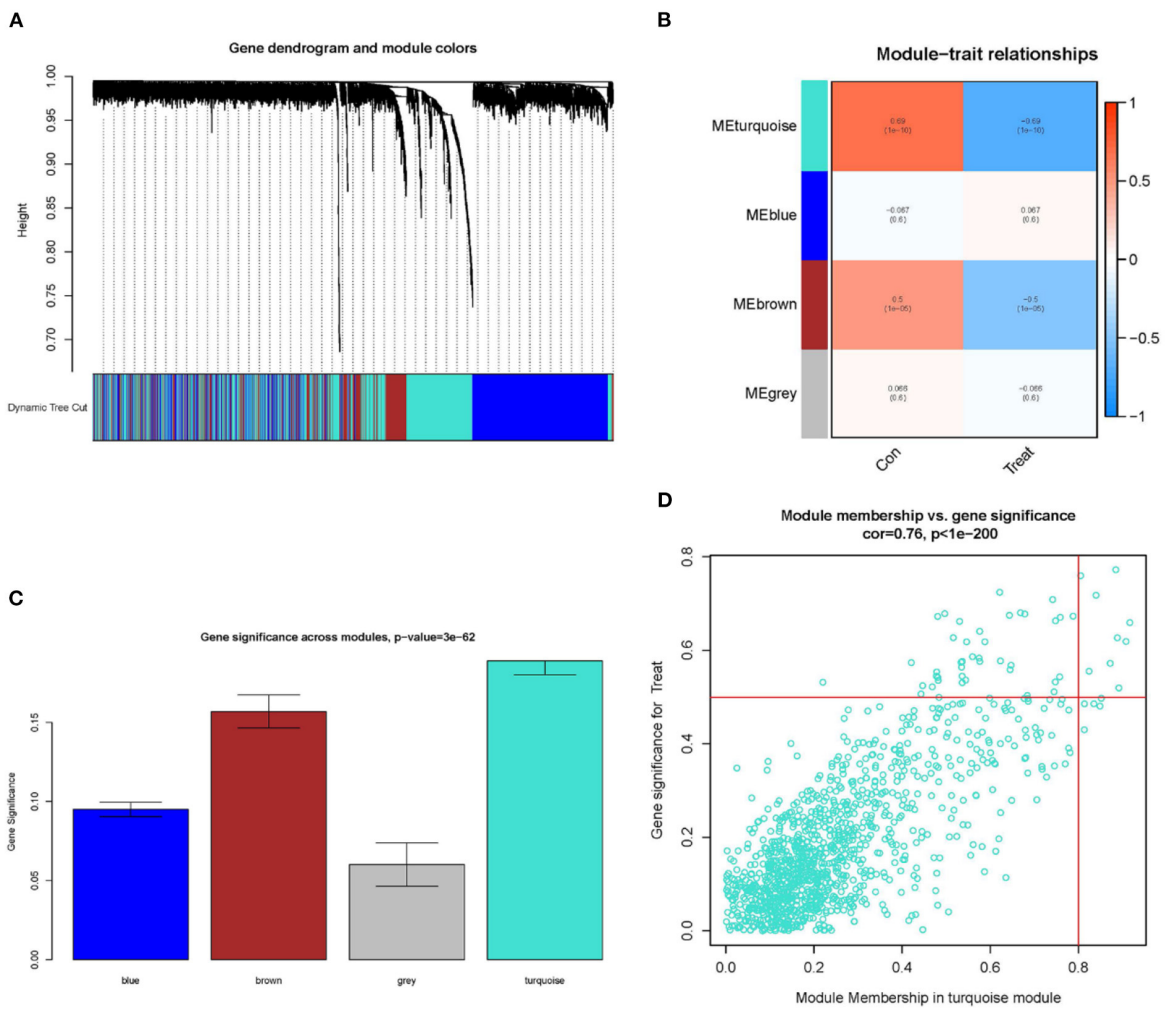
Constructing WGCNA modules and screening candidate key genes. **(A)** Gene clustering dendrogram: each branch represents a gene, and each color below represents a co-expression module. **(B)** Module-trait relationships heatmap, with turquoise modules significantly associated with controls. **(C)** Distributing gene significance in each module. **(D)** In the turquoise module, a scatter plot of gene module members vs. gene significance is shown, GS > 0.5 and MM > 0.8 are candidate key genes.

To improve the data quality, the samples are clustered, missing values are filled in, outliers are removed, and normalized the data. Therefore, when the optimal soft threshold β = 3 is selected after clustering, the constructed network is more like the scale-free network (Figures 1A,B). Subsequently, a topological overlap matrix was derived. Using dynamic hybrid shearing, we obtained gene modules, which were then clustered to create four gene modules (Figure 2A). A heatmap was used to show the correlation of the above modules with clinical traits in the control group and the cardiomyopathy group and genes' importance in each module. The strongest correlation (cor) among them was found between the turquoise module and the control group (cor = 0.69; P = 1e-10) and the highest gene importance within the turquoise module (Figures 2B,C). Lastly, according to the scatter plot, between GS and MM in the turquoise module have a strong correlation (cor = 0.76; P = 1e-200). Under the screening conditions of GS > 0.5 and MM > 0.8, 9 candidate key genes belonging to the turquoise module were obtained then used for subsequent analysis (Figure 2D, Supplementary File 4).

### Screening of differential genes and identification of hub genes

**Figure 3:**
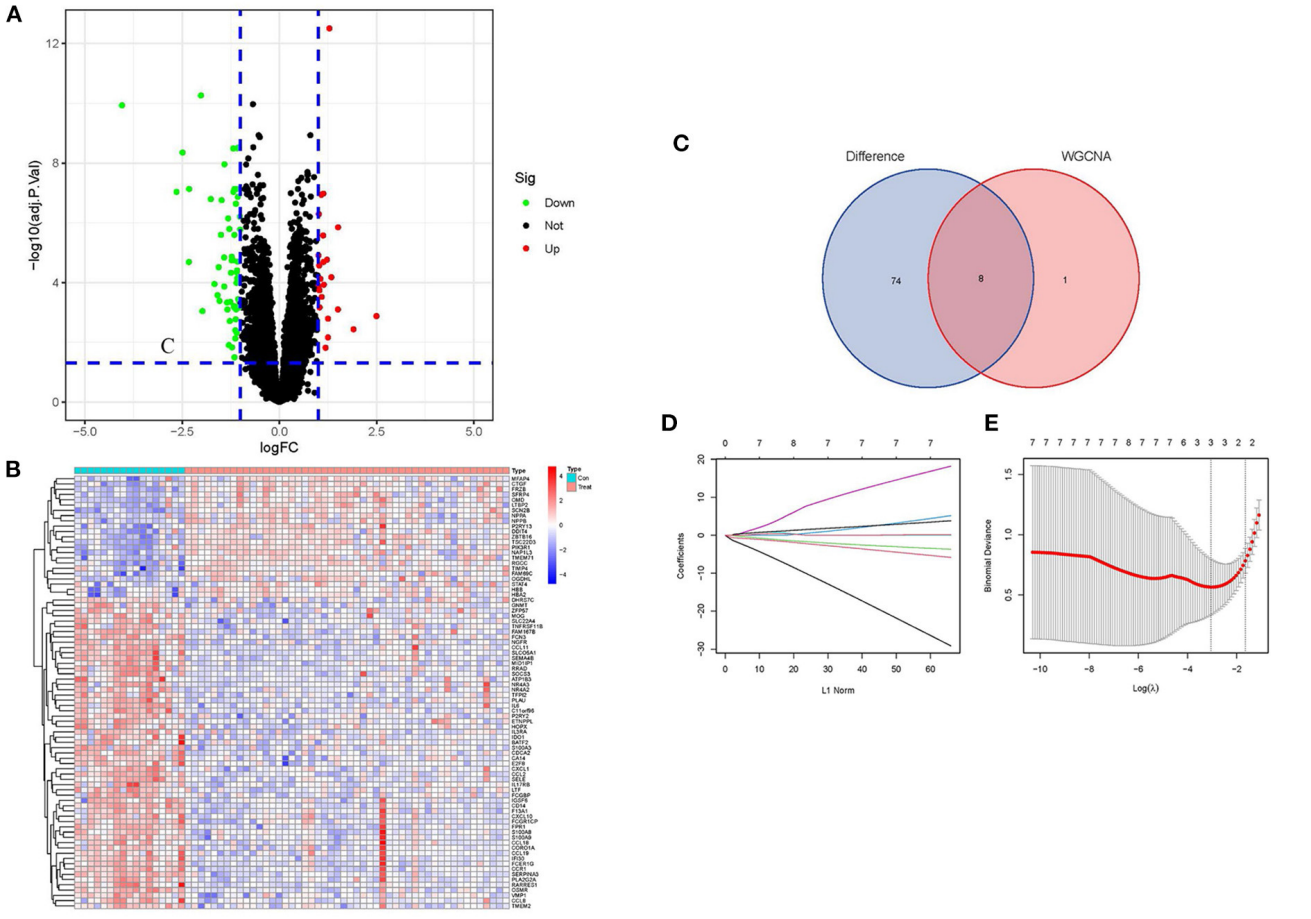
Screening of DEGs and identification of Hub genes. **(A)** Volcano plots of DEGs expression in control and cardiomyopathy groups. **(B)** Heatmap of DEGs expression in cardiomyopathy and control groups. **(C)** Venn diagram of the DEGs' intersection and candidate genes of the turquoise module. **(D)** Map of the regression coefficients of the eight genes in LASSO model. **(E)** Three hub genes screened by 10-fold cross-validation in the LASSO regression model.

Based on the DEGs' screening criteria (log fold change (FC) > 1 and adjusted-*P* < 0.05), a total of 82 DEGs were obtained (Supplementary File 3). DEGs expression in the samples was displayed in volcano plots and heatmaps (Figures 3A,B). By intersecting the DEGs with the nine candidate key genes from the turquoise module, we were able to get eight intersection genes (Figure 3C, Supplementary File 4). Finally, we performed LASSO regression analysis for the intersection genes, and the final three hub genes were identified as follows: *CD14, CCL2*, and *SERPINA3* (Figures 3D,E, Supplementary File 4).

### Identification of the expression level of the hub gene and its diagnostic value

**Figure 4:**
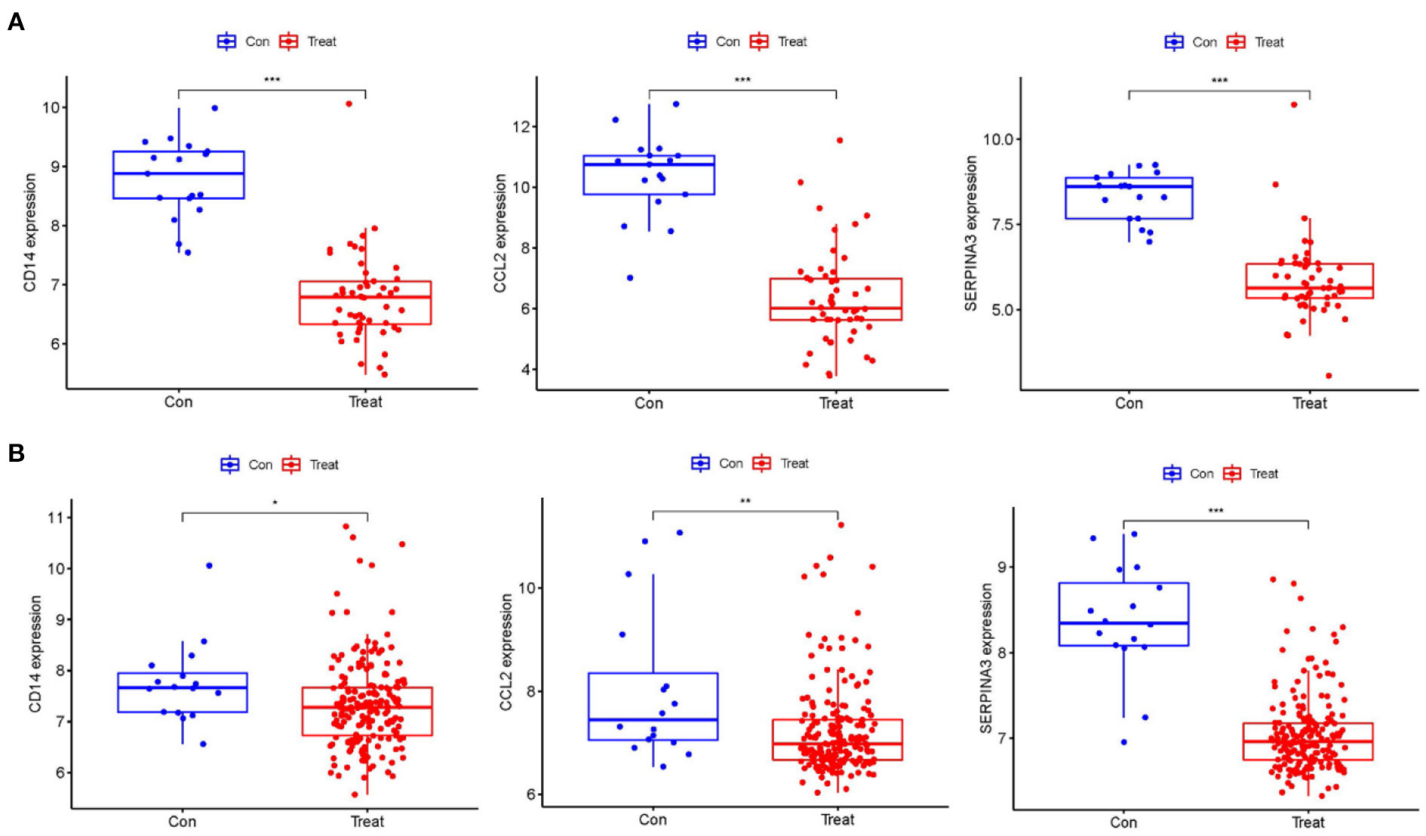
Identification of the expression level of the Hub gene. **(A)** The expression levels of hub genes, *CD14, CCL2* and *SERPINA3* in the training datasets were significantly lower in the cardiomyopathy group than in the control group. **(B)** The hub gene expression was verified in the large sample validation dataset (GSE5406), and the expressions of *CD14, CCL2* and *SERPINA3* in the cardiomyopathy group were significantly lower than those in the control group, of which *SERPINA3* had the most significant difference. “***”, “**”, “*” represent *P* < (0.001, 0.01, 0.05).

**Figure 5:**
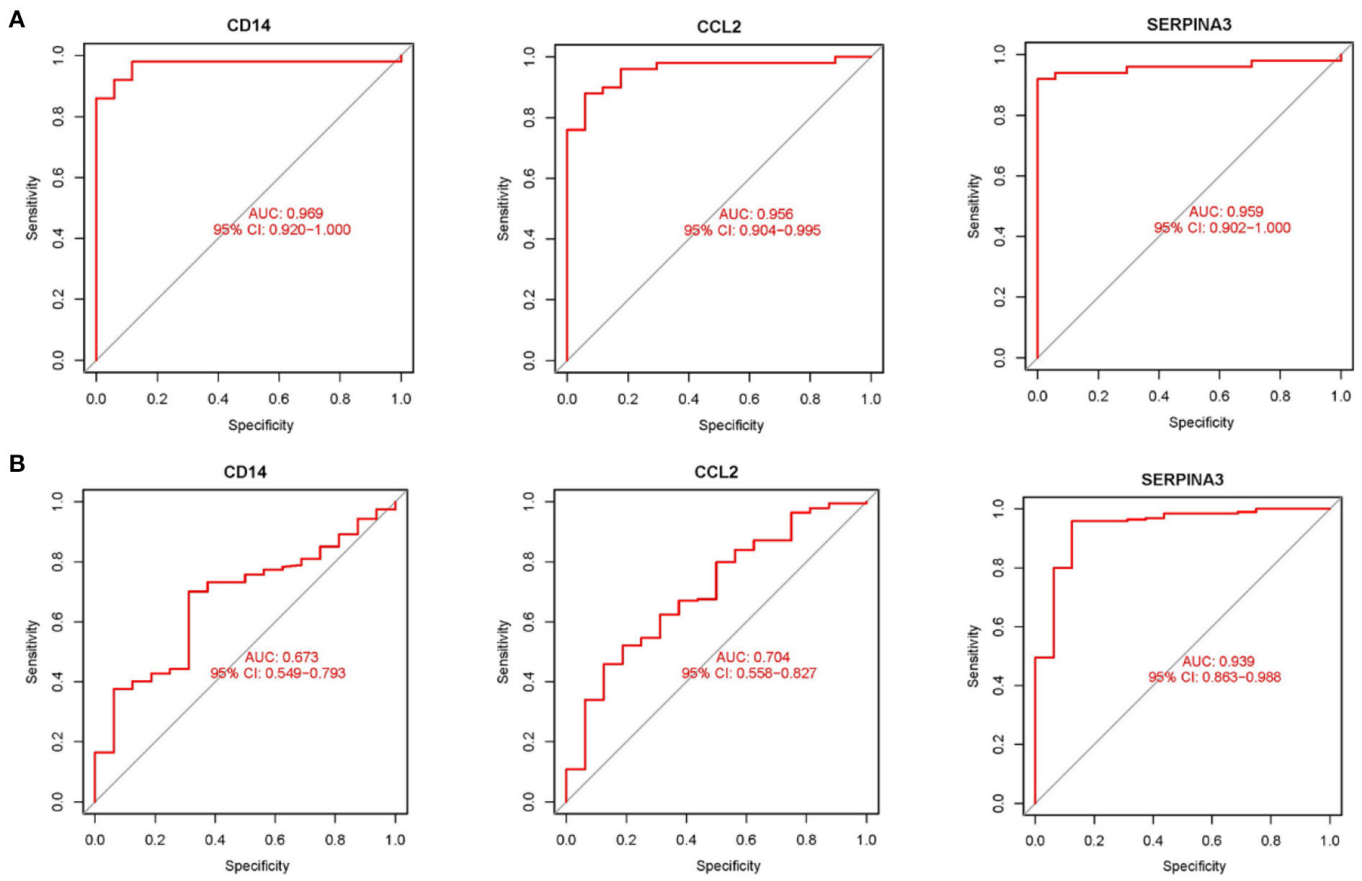
Validation hub genes are used as marker genes **(A)** Diagnostic ability of hub genes in the training datasets. The area under ROC curve (AUC) was used to evaluate the discriminating ability of hub gene in cardiomyopathy and control groups. **(B)** The validation results of hub gene in the large sample validation dataset (GSE5406) were similar to those of the training datasets.

Using boxplots, the expression levels of the three hub genes were determined. In the training dataset, the cardiomyopathy group's expression levels of *CD14, CCL2*, and *SERPINA3* were significantly lower than the ones in the control group. P values were all <0.001 (*P* < 0.001) (Figure 4A). Then, in a separate large-sample validation dataset, we further validated the expression levels of these three hub genes (GSE5406), and the results were like those of the training group, *CD14* (*P* < 0.05), *CCL2* (*P* < 0.01), and *SERPINA3* (*P* < 0.001). The expression difference of SERPINA was most significant in the validation group (Figure 4B). Our next step was to assess the precision of the three hub genes as markers for discriminating between cardiomyopathy and control groups using ROC curves. In the training datasets, the area under the ROC curve (AUC) values of *CD14, CCL2*, and *SERPINA3* genes were all > 0.95, indicating that these genes have high diagnostic values as marker genes (Figure 5A). In the validation dataset, the AUC values of the three genes *CD14, CCL2*, and *SERPINA3* were 0.673, 0.704, and 0.939, respectively. Data from the large sample validation dataset indicated that all three genes could be used as marker genes, with *SERPINA3* having the highest diagnostic sensitivity (Figure 5B).

### Functional enrichment analysis of DEGs

**Figure 6:**
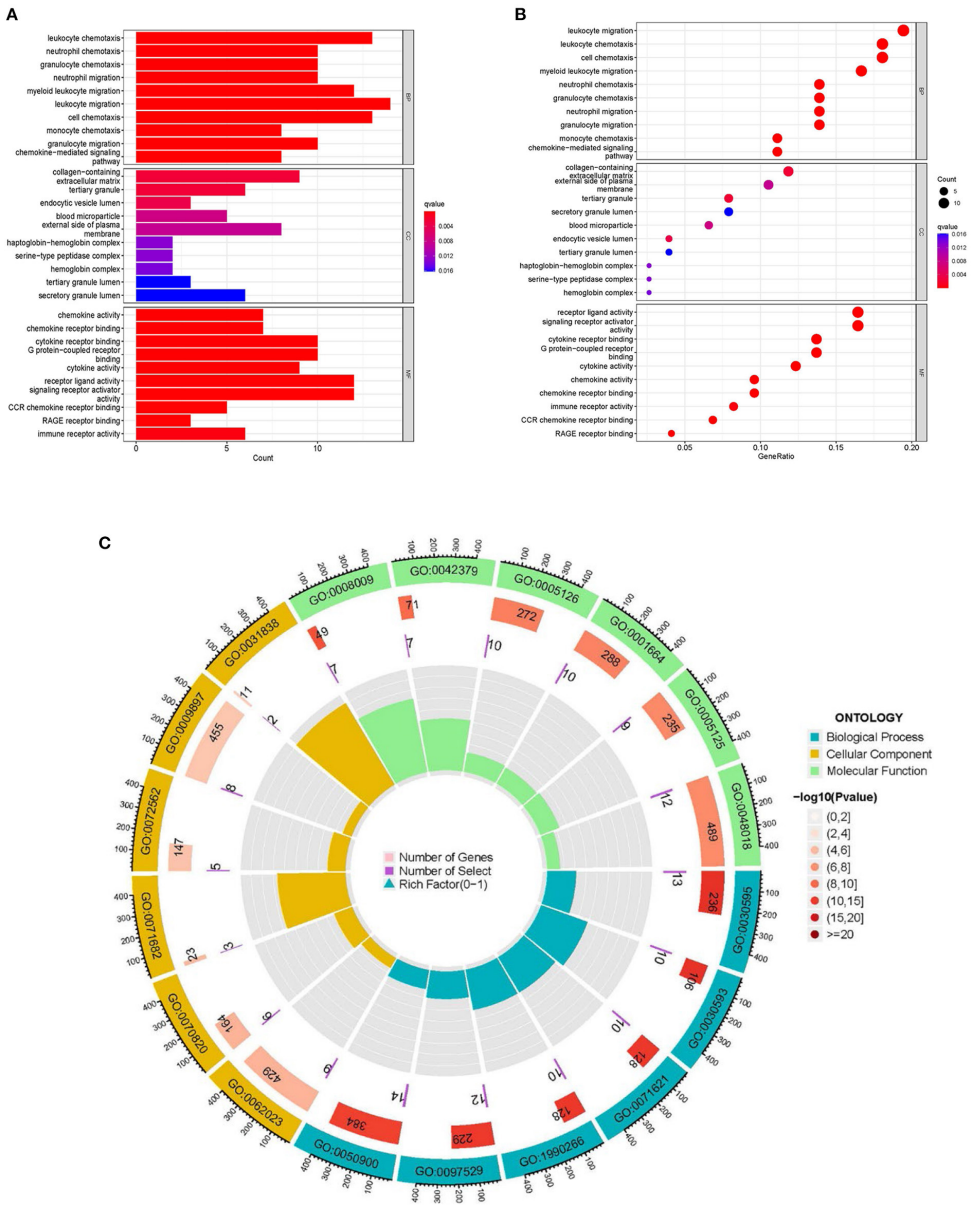
GO enrichment analysis of DEGs. **(A)** The histogram of GO enrichment analysis; the redder the color, the more significant the enrichment. **(B)** Bubble diagram representing GO enrichment analysis; the size of bubbles represents the number of enriched genes, and the redder the color of the bubbles, the more significant the enrichment is. **(C)** The first circle indicates that BP, CC, MF are represented by different colors and the top six enriched GO:ID are taken. The second circle represents the number of genes in different GO:ID genome backgrounds, where different colors represent the significant degree of DEGs enrichment. The third circle represents the number of genes enriched by DEGs. The fourth circle represents the proportion of genes.

**Figure 7:**
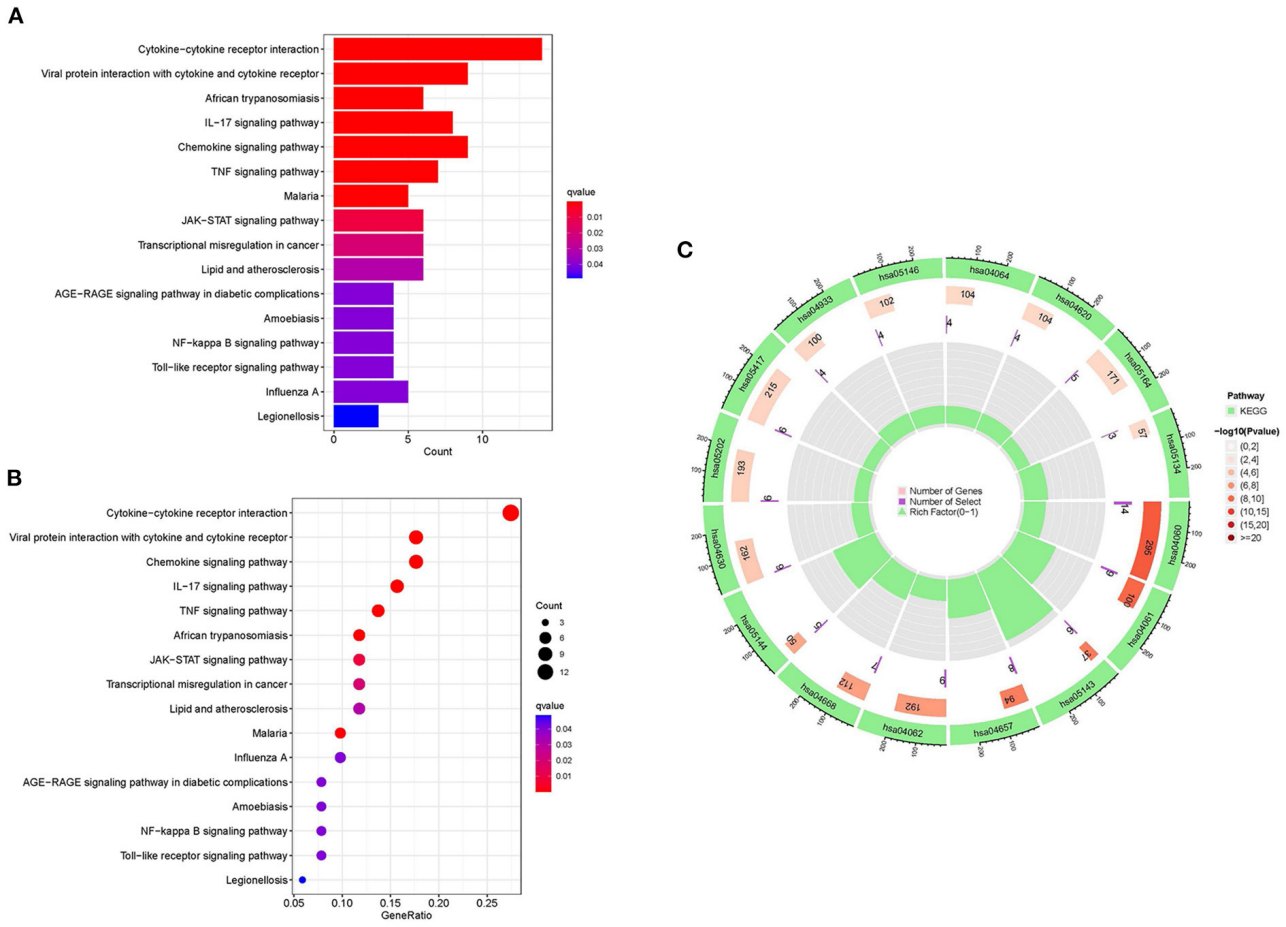
KEGG enrichment analysis of DEGs. **(A)** The histogram of KEGG enrichment analysis; the redder the color, the more significant the enrichment. **(B)** Bubble diagram representing KEGG enrichment analysis; the size of bubbles indicates the number of enriched genes, and the redder the color of bubbles, the more significant the enrichment is. **(C)** The first circle represents the enriched KEGG:ID. The second circle represents the number of genes in different KEGG:ID pathway backgrounds, where different colors represent the significant degree of DEGs enrichment. The third circle represents the number of genes for which DEGs are enriched in the pathway. The fourth circle represents the proportion of genes.

KEGG enrichment analysis and GO were done to comprehend the related signaling pathways and important biological functions involved in DEGs (Supplementary File 5). Biological functions are mainly enriched in defense and inflammatory processes, such as leukocyte chemotaxis and migration. In terms of cellular composition, it is mainly enriched in the extracellular matrix. The molecular functions are primarily enriched in receptor activities, including G protein-coupled receptors, signaling receptor activators, etc. (Figures 6A–C). The KEGG signaling pathway enrichment analysis revealed that DEGs were mainly abundant in inflammation-related signaling pathways, such as chemokine, cytokine-cytokine receptor interaction and IL-17 signaling pathways (Figures 7A–C). These findings uncovered cellular mechanisms and abnormal signaling pathways implicated in the development of cardiomyopathy.

### Immune signature gene set enrichment analysis

**Figure 8:**
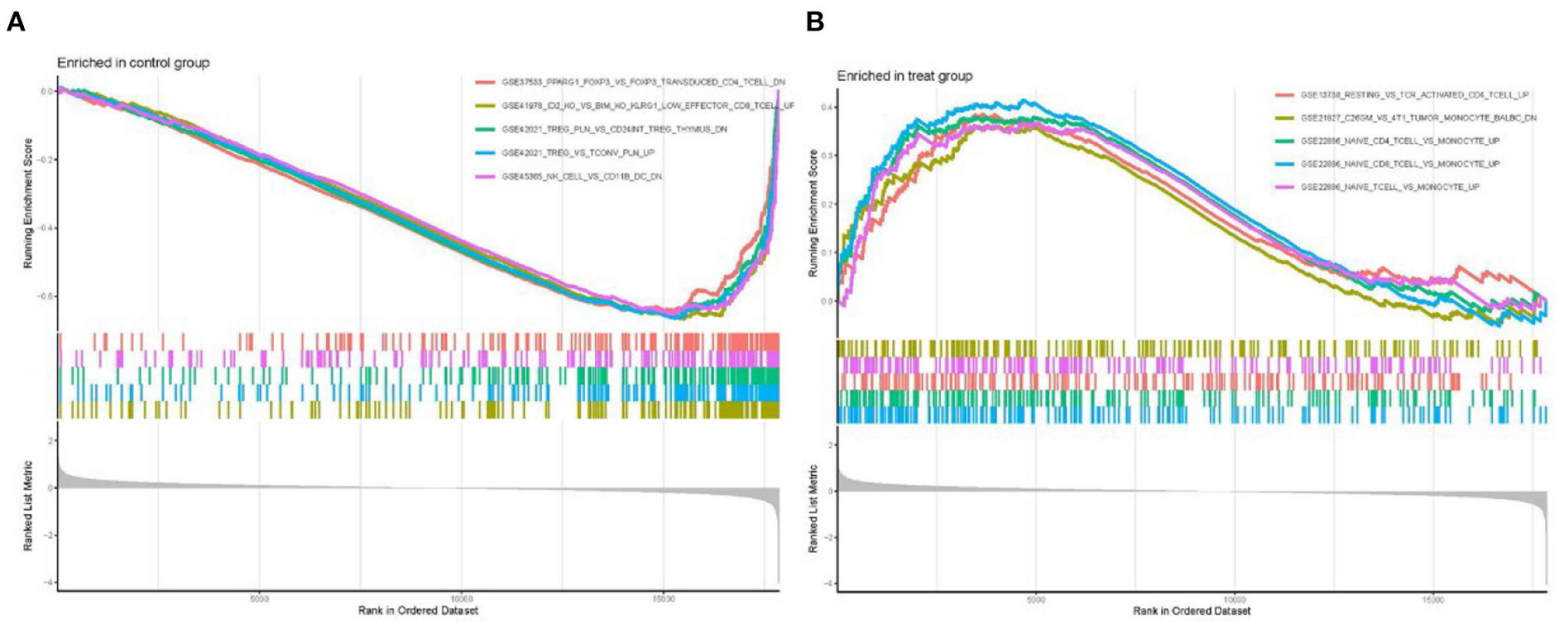
Enrichment of GSEA immune signature gene set. **(A)** Immune gene set scores in the control group. **(B)** Immune gene set scores in the cardiomyopathy group.

GSEA enrichment analysis was performed on all differential gene files using the immune signature gene set in MsigDB database to identify the underlying immune-related mechanisms during cardiomyopathy progression. A total of 1,281 gene sets were enriched (q <0.05, Supplementary File 6). We showed the top five most significantly enriched gene sets, among which the gene sets CD4+ T cells, CD8+ T cells, and naive T cells have high enrichment scores in the cardiomyopathy group but low scores in the control group. In contrast, natural killer cells and regulatory T cells were highly enriched in the control group. These findings demonstrated the importance of adaptive immune cell in the progression of cardiomyopathy, while regulatory immune cells and innate immune cells may play a role in heart protection (Figures 8A,B).

### ssGSEA analysis of immune cell infiltration and its correlation with hub genes

**Figure 9:**
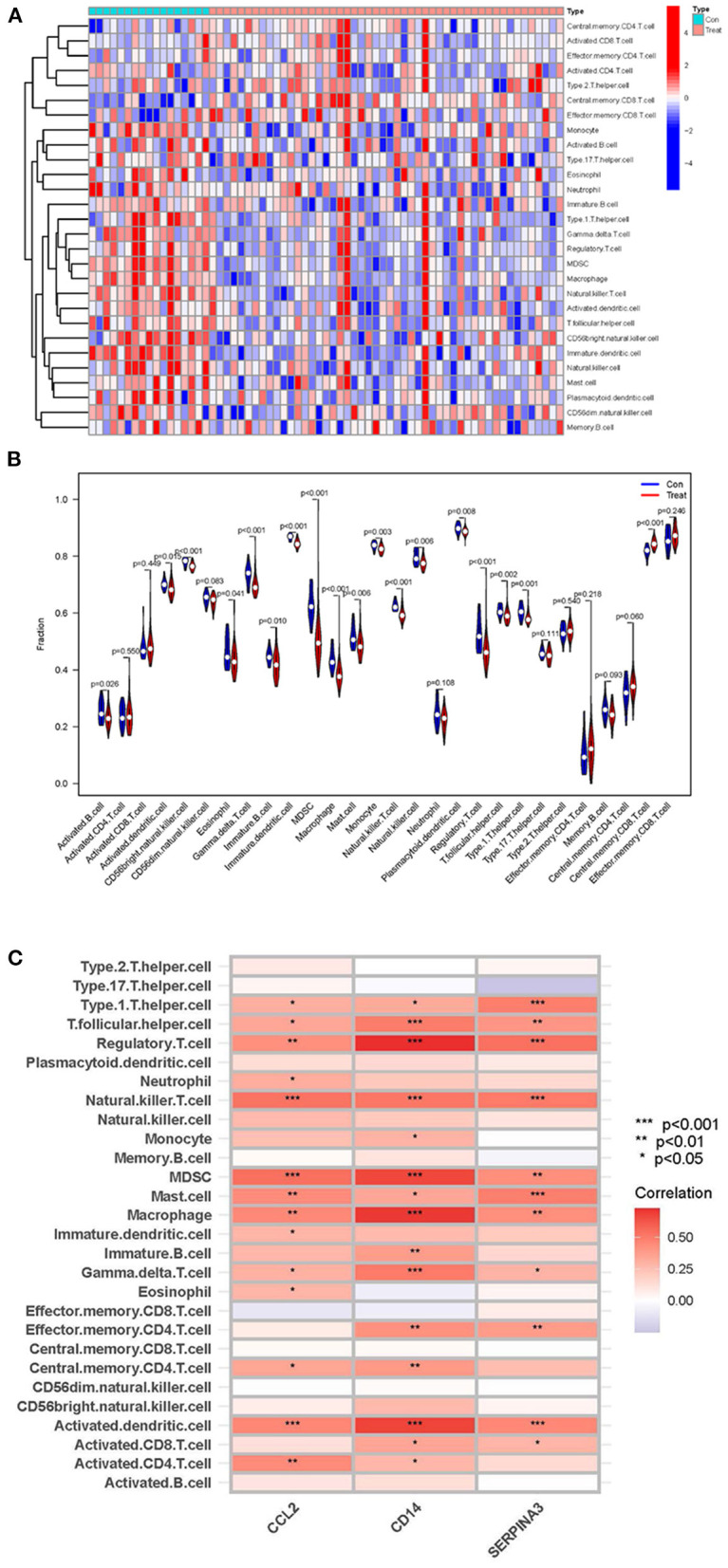
ssGSEA analysis of immune cell infiltration and its correlation with hub genes. **(A,B)** Heatmaps and violin plots showing the differences and distribution of 28 immune cells in cardiomyopathy and control groups. **(C)** The relation between immune cell infiltration and three hub genes; the redder the color, the more significant the difference. “***”, “**”, “*” represent *P* < (0.001, 0.01, 0.05).

By using ssGSEA analysis, we first compared immune cell infiltration between cardiomyopathy and control groups. The distribution of the 28 immune cells in the expression profile samples is shown in Figure 9A and Supplementary File 7. The immune cell infiltration analysis results showed that regulatory T cells, myeloid-derived suppressor cells, type 1 helper T cells, and macrophages were lower in the cardiomyopathy group than in the control group (Figure 9B). Then we evaluated the association of 28 immune cells with hub genes, among which regulatory T cells were associated with *CD14* (*P* < 0.01), *CCL2* (*P* < 0.001) and *SERPINA3* (*P* < 0.001), activated dendritic cells, myeloid-derived suppressor cells with *CD14* (*P* < 0.001), *CCL2* (*P* < 0.001) and *SERPINA3* (*P* < 0.01), natural killer cells with *CD14* (*P* < 0.001), CCL2 (*P* < 0.001) and SERPINA3 (*P* < 0.001), macrophages were positively correlated with *CD14* (*P* < 0.01), *CCL2* (*P* < 0.001) and *SERPINA3* (*P* < 0.01) (Figure 9C). These findings may demonstrate the critical role of regulatory and innate immune cells in heart protection.

## Discussion

Recently, WGCNA analysis has replaced DEGs-based screening approaches because of their deficiencies. For example, traditional methods are only used to study a small number of datasets, and the correlation between genes is “one size fits all,” which is prone to overlook essential regulatory core molecules in the regulatory process of biological systems. By contrast, the core molecules associated with clinical traits can be identified based on the weighted gene co-expression network (21). LASSO provides a dimensionality reduction impact, as a regression analysis approach, compared to classical logistic and Cox regression (17). In this study, we analyzed data by WGCNA to determine candidate genes that are strongly related to the clinical traits of the cardiomyopathy group and the control group. Following, we intersected DEGs with the candidate key genes to identify divergent and highly correlated intersecting genes between the two groups. Eventually, LASSO regression analysis identified three hub genes: *CD14, CCL2*, and *SERPINA3*. The expression level of these three hub genes was significantly lower in the cardiomyopathy group when compared to the control group. The AUC represents its sensitivity and accuracy as a marker gene. Among them, *SERPINA3* had the highest diagnostic efficiency and was the most strikingly differentially expressed gene. In addition, it is worth noting that previous research has focused on cardiomyopathies of a single etiology, which can improve the understanding of the pathological mechanisms of specific diseases, but may lack the exploration of common pathological mechanisms involved in cardiomyopathies of different etiologies. However, our current study is innovatively investigation to combine cardiomyopathy with varied etiologies which examined common hub genes and their diagnostic ability as marker genes and how they contribute to the immune infiltration pattern of various cardiomyopathies. Especially, we have assessed the diagnostic ability and accuracy not only in the training dataset, but also in the large sample validation dataset as well. In this way, our results are very reliable and trustworthy.

The innate immune system functions as an “early warning system,” allowing the host to differentiate between non-self and self accurately and quickly. It is activated by “pattern recognition receptors” found on many cells, including cardiomyocytes. Cardiomyocytes express various pattern recognition receptors like Toll-like and *CD14* receptors. The cardiac innate immune system relies on these pattern recognition receptors to respond to various forms of myocardial injury (22, 23). The *CD14* receptor encoded by the *CD14* gene is a leucine-rich receptor mainly expressed on the surface of natural cells, especially monocytes/macrophages. It can also act as a receptor in a soluble form (sCD14) on cells that do not express CD14 on their surface, such as dendritic cells (24). It plays an important protective role as a pattern recognition molecule to recognize a variety of inflammatory mediators (25). One of the CC chemokines, called monocyte chemoattractant protein-1 (*CCL2; MCP-1*), is important for the migration of monocytes, memory T cells, and natural killer cells (26, 27). Several studies have shown that monocytes/macrophages are involved in the pathogenesis and occurrence of cardiovascular disease by retaining and activating *CCL2*. As in ischemic cardiomyopathy, *CCL2* has a persistent chronic expression (28). In our study, the expression level was lower compared to that of the control group, and it may be used as a target molecule to distinguish chronic cardiomyopathy from normal myocardium, or its dysregulation promotes cardiomyopathy development. Unquestionably, this needs to be verified in large samples and further experiments.

As a member of the serine protease inhibitor superfamily, *SERPINA3*, also known as α1-antichymotrypsin, is implicated in oxidative stress, apoptotic cell death and inflammatory responses (29). The *SERPINA3* gene expression is regulated by cytokines such as IL1 and IL6. In addition, the *SERPINA3* gene expression as part of inflammatory responses can regulate immune cells via mast cell chymotrypsin, leukocyte elastase, and neutrophil cathepsin G (30). Inadequate regulation of *SERPINA3* can result in prolonged or excessive cathepsin G activity, eventually causing tissue injury (31). Studies have shown that *SERPINA3* is associated with systemic inflammation and oxidative stress. In stable heart failure, excess levels can have detrimental effects on cardiac function and increased mortality or cardiac accidents. *SERPINA3* may be as well utilized as a heart failure predictive biomarker with great potential (32, 33). Consistent with previous studies, our study showed that the number of samples expressing *SERPINA3* in the cardiomyopathy group was significantly greater compared to the control group. However, in comparison with the control group, the expression level was significantly lower, so this specifies that the dysregulation or imbalance of *SERPINA3* gene expression is involved in heart disease progression in those with cardiomyopathy.

Furthermore, the difference in *SERPINA3* expression levels between the two groups, and its ability to serve as a marker gene to distinguish the cardiomyopathy group from the control group, was significantly greater than that of *CD14* and *CCL2*. Thus, these merits further investigation into the diagnostic and therapeutic potential of SERPINA3 in cardiomyopathy. Most importantly, we found that both *CCL2* and *CD14*, which are closely related to monocytes/macrophages, and *SERPINA3*, regulated by cytokines IL1 and IL6, appear to be involved in the innate immune response. We conjecture that their dysregulation and imbalance may contribute to the cardiomyopathy progression. To better understand this finding, we further performed an enrichment analysis of the data to explore the immune-related pathways involved.

GO enrichment analysis of DEGs exhibited that the biological functions were primarily concentrated in the immune and inflammatory response. However, this is consistent with our previous presentation, which stated that after cardiac injury triggers inflammatory and immune responses, it promotes tissue healing and remodeling by activating compensatory mechanisms. Though, remodeling and inflammation become chronic over time, decreasing cardiac function and heart failure (34). Cardiac fibrosis occurs in the progression of different etiologies of cardiomyopathy, and enrichment analysis of cellular component shows that the extracellular matrix is closely related to fibrosis (35). The molecular function enrichment of signaling receptor activator activity and G protein-coupled receptors represent central physiological functions involved in cardiomyocyte growth, metabolism, and functional regulation. The KEGG signaling pathway showed that DEGs were predominantly enriched in chemokine, cytokine-cytokine receptor interaction and IL-17 signaling pathway. Cytokines participate in the coordination of the immune system during the host's defense, and their release after organism injury triggers innate and adaptive immunity (36). Cardiomyopathy may be triggered by cytokines, which are believed to have a role in the pathophysiology of several forms of cardiac dysfunction (37, 38). Th17 cells are a distinct subset of CD4+ T helper cells that primarily produce IL-17 cells, which link adaptive and innate immune responses. Activating IL-6, transforming growth factor-β and IL-1 in a pro-inflammatory cytokine milieu enables naive CD4+ T cells to differentiate and prime Th17 cells (36). Studies have found that the Th17/CD4+ T cells' imbalance may play a crucial part in the process of myocardial injury, and the higher the proportion of Th17 cells, the more obvious the decrease in cardiac function. However, the high expression of IL-17 can aggravate the induction of ventricular hypertrophy and myocardial fibrosis, leading to ventricular remodeling (39, 40). Finally, GSEA enrichment analysis of expression profile files showed that CD8+T cells, CD4+T cells and naive T cells had high enrichment scores in the cardiomyopathy group, while natural killer cells and regulatory T cells had high enrichment scores in the control group. CD4+ T cell subsets now include TH1 cells, TH2 cells, TH17 cells and regulatory T cells. Regulatory T cells, expressing CD25 and the transcription factor FoxP3, have immunomodulatory properties that help suppress inflammation and autoimmune diseases (41). As an adaptive immune response, TH1, TH2 cells, and the previously described Th17 cell subtype have been implicated in the progression of myocardial disease in numerous studies (4, 5). These findings highlighted the crucial significance of genes associated with adaptive immune cells in the incidence and development of cardiomyopathy, while regulatory and innate immune cells may be involved in the protection of the heart.

Finally, we analyzed the infiltration of 28 immune cells in the cardiomyopathy group and the non-cardiomyopathy control group using the ssGSEA algorithm. The findings indicated that in the cardiomyopathy group, myeloid-derived suppressor cells, regulatory T cells, type I helper T cells, and macrophages were lower compared to the control group. Further analysis of the most relevant immune infiltrating cells involved in the three hub genes showed that regulatory T cells, myeloid-derived suppressor cells, activated dendritic cells, macrophages and natural killer cells were most closely related to the hub genes. According to many scholars' studies, it demonstrated that that regulatory T cells, myeloid-derived progenitor cells, monocytes/activated macrophages, or dendritic cells can play a suppressive regulatory function in the myocardial disease development (42, 43). These results are consistent with our study. Based on the above analysis, we further proved that the control group was more involved in the innate immune response, while the adaptive immune response played an important role in the cardiomyopathy group. Our studies suggest that the heart may protect itself through regulatory T cells and innate immune cells; however, dysregulation and imbalance of innate immune cells and activation of adaptive immune responses are involved in cardiomyopathy disease progression in patients. These findings deepen our understanding of the immune system's role in cardiomyopathy progression. It can better guides scholars for clinical drug development and might help efforts to develop more targeted specific immunotherapy for cardiomyopathy.

Because of its limitations, this study can be validated further in prospective and larger-sample studies to eliminate invasive diagnostics and to give a guideline for early detection and focused medication development for cardiomyopathy of diverse etiologies. The reasons are as follows: Firstly, this study was retrospective and did not include all etiologies of cardiomyopathy, such as HCM, so a larger prospective study is needed to validate our conclusions. Secondly, the data set sample of the training group in this study is still small, so the accuracy of the assessment and prediction of the disease Hub gene can be improved by increasing the sample size. In addition, there are some other limitations that need to be highlighted. For example, further experimental validation using animal models of cardiomyopathy or tissue samples from human cardiomyopathy patients is needed. Moreover, the present study can only support the correlation analysis between cardiomyopathy and immune cells and between Hub genes and immune cells, but cannot reveal the cause-and-effect relationship. Finally, the subjects in this study may differ in terms of geography, race, living environment, genetic variation and susceptibility to cardiomyopathy. All these factors may have an impact on the study of cardiomyopathy.

In conclusion, with the intersection of multiple cardiomyopathy gene sets with different etiologies and WGCNA analysis and LASSO regression analysis, we screened out a key module (turquoise module) and three hub genes involved in cardiomyopathy progression (*CD14, CCL2*, and *SERPINA3*). After that, we combined bioinformatics analyses of GO, KEGG, GSEA, and ssGSEA. This study demonstrated differential expression of hub genes and their diagnostic ability as marker genes, but it also contributed to the common immune infiltration pattern of various cardiomyopathies and provided insights into the underlying immunomodulatory mechanisms.

## Data availability statement

The datasets presented in this study can be found in online repositories. The names of the repository/repositories and accession number(s) can be found in the article/Supplementary material.

## Author contributions

BH was engaged to write and conceptualize the original draft and were responsible for methodology. L-PQ, C-YC, D-YY, WY, Q-JW, ZZ, and X-NH were responsible for software. LL was responsible for reviewing and editing. The published version of the work has been reviewed and approved by all authors.

## Funding

This study was sponsored by funds from the Chinese National Natural Science Foundation of China (81560076), Guangxi Natural Science Foundation Youth Program (2018JJB140358), Middle-aged and Young Teachers in Colleges and Universities in Guangxi Basic Ability Promotion Project (2021KY0534), and The First Batch of High-level Talent Scientific Research Projects of the Affiliated Hospital of Youjiang Medical University for Nationalities in 2019 (R20196316).

## Conflict of interest

The authors declare that the research was conducted in the absence of any commercial or financial relationships that could be construed as a potential conflict of interest.

## Publisher's note

All claims expressed in this article are solely those of the authors and do not necessarily represent those of their affiliated organizations, or those of the publisher, the editors and the reviewers. Any product that may be evaluated in this article, or claim that may be made by its manufacturer, is not guaranteed or endorsed by the publisher.
